# Dietary Patterns, Cardiometabolic and Lifestyle Variables in Greeks with Obesity and Metabolic Disorders

**DOI:** 10.3390/nu14235064

**Published:** 2022-11-28

**Authors:** Charalampia Amerikanou, Stamatia-Angeliki Kleftaki, Evdokia Valsamidou, Chara Tzavara, Aristea Gioxari, Andriana C. Kaliora

**Affiliations:** 1 Department of Nutrition and Dietetics, School of Health Science and Education, Harokopio University, 70 El. Venizelou Ave, 17676 Athens, Greece; 2 Department of Nutritional Science and Dietetics, School of Health Science, University of the Peloponnese, Antikalamos, 24100 Kalamata, Greece

**Keywords:** dietary patterns, metabolic disorders, obesity, dyslipidemia, insulin resistance, anthropometrics, mental health, plant-based diet, Mediterranean diet, Western diet

## Abstract

There is considerable evidence that some dietary patterns contribute to obesity and metabolic disorders but there is less data on diet’s association with different health parameters. We investigated the interaction between different dietary patterns and anthropometric, biochemical, lifestyle, and psychological health parameters in a Greek population with obesity and metabolic disorders. To the best of our knowledge, this is the first study in Greece with a thorough and holistic approach in analyzing such relationships. For assessing food patterns, revealing underlying structures, and reducing the number of variables we applied exploratory factor analysis (EFA). Principal Component Analysis was chosen as the extraction method using Varimax rotation, and three regression sets were computed. The study involved 146 Greek metabolically unhealthy obese adults, both men and women. Our cohort was categorized into four dietary patterns: “Western type diet”, “Mediterranean-like diet”, “Healthy diet”, and “Animal meat and sauces diet”. Dietary patterns characterized by a high consumption of energy-dense and animal-derived foods were positively associated with anthropometric and biochemical parameters related to metabolic disorders. Plant-based, healthier dietary patterns, on the other hand, were associated with better biochemical and mental health profiles among metabolically unhealthy obese individuals.

## 1. Introduction

Nowadays, chronic metabolic disorders are a growing public health concern, especially obesity and diabetes [1]. The prevalence of overweight adults exceeded 1.9 billion in 2016, with a great proportion (650 million) being obese [2]. Similarly, the International Diabetes Foundation (IDF) reported that prevalence of diabetes in 2019 was 463 million, and this is expected to increase further and reach 700 million by 2045 [3]. Apart from type 2 diabetes mellitus (T2DM), obesity has been associated with many other metabolic disorders, such as cardiovascular diseases (CVDs), non-alcoholic fatty liver disease (NAFLD), several types of cancers and, recently, it was linked with COVID-19 mortality [4]. Although body weight is influenced by both genetic and environmental factors, the increased prevalence of adiposity globally suggests that urbanization, followed by food marketing and access to refined and energy-dense food items, along with reduced physical activity, are the main drivers of obesity [5].

Since obesity has been associated mainly with diet quality and unhealthy dietary choices, rather than single foods or nutrients, dietary patterns analysis is one of the best approaches to examine the influence of overall diet, by including the interactive effect of individual items, macro- and micro- nutrients, and bioactive compounds. Furthermore, it offers the opportunity to associate diet with several parameters related to metabolic disorders, such as anthropometric and cardiometabolic markers [6,7]. Moreover, dietary patterns responsible for obesity could be used as a tool for its prevention [8]. The effects of several dietary patterns on obesity and related metabolic disorders have been investigated throughout the years. Most studies evidence that diets with high consumption of plant foods, such as the Mediterranean diet, are valuable in the prevention of obesity, whereas dietary patterns with a high intake of red meat and refined grains, such as the Western pattern, increase the risk of obesity and related comorbidities [9].

It is well established that several dietary patterns contribute to obesity and metabolic disorders. Nevertheless, a wide range of anthropometric, biochemical, and lifestyle parameters have seldom been linked to diets among Greeks. Therefore, in this study, we aimed to explore the interaction between different dietary patterns in a Greek population with obesity and metabolic disorders with several related cardiometabolic and lifestyle parameters. More specifically, apart from anthropometric and biochemical indices, we addressed the associations with lifestyle, physical activity, and physical and psychological health parameters as well.

## 2. Materials and Methods

### 2.1. Study Design

This observational study used the baseline data from a three-month intervention that investigated the effect of a mushroom-based snack on markers related to obesity and metabolic disorders [10]. The inclusion criteria were adult age, central obesity, and metabolic disorders, including dyslipidaemia, glucose intolerance, insulin resistance and hypertension. Non-eligible subjects were pregnant and lactating females, patients with thyroid disease, or with abuse of alcohol, or with known psychiatric or mental disorders. The Ethics Committee of Harokopio University approved the protocol of the study (ID protocol: 62/03-07-2018), which was in line with the Helsinki Declaration and the Data Protection Act 1998. Additionally, the trial acquired registration with clinicaltrials.gov (ID Number: NCT04081818). Patient recruitment took place in Harokopio University of Athens (Greece) between 2020 and 2021. All subjects were informed in detail about the study before giving their signed consent.

### 2.2. Anthropometric Measurements

Body weight (BW), height, body composition i.e., body fat (BF), fat free mass (FFM), and total body water (TBW), as well as waist and hip circumferences (WC, HC), and waist-to-hip ratio (WHR), were measured. Body weight was recorded to the nearest kg using a flat scale, early in the morning with light clothing and without shoes. Accordingly, height was measured to the nearest cm using a stadiometer (Seca Mode 220, Hamburg, Germany). Body mass index (BMI) was calculated as BW (kg)/Height^2^ (m^2^). To estimate body composition (BF, FFM, TBW) bioelectrical impedance analysis (Tanita BC-418, Tokyo, Japan) was performed. Body circumferences (WC, HC, WHR) were measured with a non-stretch, but flexible tape, on minimal clothing.

### 2.3. Biochemical Parameters

Blood samples, of 20 mL, were collected after an overnight fast and were centrifuged at 3000 rpm for 10 min at 20 °C for serum isolation. Serum biochemical markers were measured with an automatic biochemical analyser (Cobas 8000 analyser, Roche Diagnostics GmbH, Mannheim, Germany): glucose; insulin; total cholesterol (TC); high-density lipoprotein (HDL); low-density lipoprotein (LDL); triglycerides (TG); urea; uric acid; creatinine; alanine aminotransferase (ALT); aspartate aminotransferase (AST); γ-glutamyl transferase (γ-GT); alkaline phospatase (ALP); uric acid; lactate dehydrogenase (LDH); iron (Fe); ferritin; albumin; and C-reactive protein (CRP).

Vitamin D was measured with an automated immunoassay system, Cobas e801 (Roche Diagnostics, Mannheim, Germany).

### 2.4. Physical Activity and Quality of Life Assessment

We assessed physical activity, smoking status, risk for depression, self-esteem, insomnia level and related factors of quality of life. To assess physical activity, the International Physical Activity Questionnaire Short Form (IPAQ-SF) was applied. Physical activity levels were expressed as the metabolic equivalent task in minutes per week (MET-min/week), based on the IPAQ scoring system [11]. Regarding smoking status, participants were asked about their smoking habits and were classified as smokers and non-smokers. To evaluate the risk for depression, the 10-item questionnaire Center for Epidemiologic Studies Depression Scale Revised (CESD-R-10) was used. Scoring ranged between 0 and 60, while scores ≥16 were considered as depression [12]. Self-esteem was assessed by applying the 10-item Rosenberg Self-Esteem scale that includes both positive and negative feelings about oneself [13]. Sleep quality was evaluated by the Athens Insomnia Scale (AII), consisting of 10 items on nocturnal sleep problems and daytime dysfunction [14]. Finally, the impact of health on an individual’s everyday life was assessed through the self-reported Short Form-12 (SF-12) questionnaire with two summary scores reporting on a mental (MCS-12) and a physical component (PCS-12).

### 2.5. Dietary Assesment

Dietary patterns were produced using data from a standardized semi-quantitative (69 items) food frequency questionnaire (69-FFQ) [15]. This questionnaire evaluated the long-term habitual intake of 69 food items and beverages, such as dairy, cereals, meat, fish, legumes etc. For each food item the participants were asked to choose one of the following consumption options: “never/rarely”, “1–3 times/month, 1–2 times/week, 3–6 times/week, 1 times/day,” to “≥2 times/day”. Finally, the 69 food items were divided into 25 food groups with similar nutrient profiles, as presented in Supplementary Table S1.

### 2.6. Statistical Analysis

Statistical analyses were conducted using SPSS statistical software (version 24.0). We carried out Exploratory Factor Analysis (EFA) to evaluate food patterns, disclose underlying structures and reduce the number of variables. Varimax rotation was applied as the extraction method for Principal Component Analysis (PCA). For sample adequacy the Kaiser–Meyer-/–Olkin (KMO) procedure was used. Values of 0.40 and 1.00 were set as cut-off points for factor loadings and Eigen values, respectively. Calculation of Cronbach’s alpha coefficient was used to determine internal consistency reliability. Scales with reliabilities equal to, or greater than, 0.70 were considered acceptable. Spearman’s rho correlation coefficients were used to explore the association of dietary patterns with biochemical and the other study measurements. For associations that were found to be significant, adjustments were made via linear regression analyses. Three sets of regression were computed, one with adjustment for age, gender and BMI, one with adjustment for age, gender, BMI, physical activity and smoking and the last with adjustment for age, gender, BMI, physical, smoking, current medication and the other patterns. Adjusted regression coefficients (β) with standard errors (SE) were computed from the results of the linear regression analyses. Regression analyses were conducted after having logarithmically transformed the dependent variables. Statistical significance was set at *p*-value < 0.05, and all *p*-values were two-tailed.

## 3. Results

A total of 146 Greek metabolically unhealthy obese adults, men and women, were enrolled in the dietary patterns study. The descriptive characteristics of the sample are depicted in Table 1.

For the PCA, the sampling adequacy of EFA was evaluated through a KMO of 0.72 and a significant Bartletts’ sphericity (*p* < 0.001). The analysis of the 25 food groups revealed 4 principal components/factors, each characterizing a possible dietary pattern. All factors combined explained 44.2% of the variance. Factor 1 had 10 food groups and explained 16.7% of the variance. Factor 2 had 7 food groups and explained 13.4% of the variance, factor 3 had 3 food groups and explained 7.6% of the variance, while factor 4 also had 3 food groups and explained 6.5% of the variance. All food groups had a loading of at least 0.40, except for eggs and alcohol that did not load to any of the four factors as their highest loading was 0.29. All factors had acceptable reliability, since their alpha coefficient of Cronbach was above 0.7.

The factor loadings between the 25 food groups and the 4 dietary patterns are presented in Table 2. The first pattern was dubbed as a “Western-type pattern”, which was characterized by high fat dairy, refined grains, fast food and processed meat, pies, sweets, salty snacks, soft drinks, animal and hydrogenated fats and seed oil. The second pattern, the “Mediterranean-like diet pattern” included whole grains, fish, vegetables (raw and cooked), fruit (raw, fruit juices and dried fruits), pulses and nuts. The third pattern, “Healthy pattern”, included low-fat dairy, olive oils and oil, coffee and tea. Finally, the last pattern, the “Animal meat and sauces pattern”, encompassed red meat, poultry and sauces.

The results of the correlation analysis of the dietary patterns with the parameters examined in our study are presented in Table 3. More specifically, Table 3 depicts correlations of dietary patterns with anthropometric measurements, with biochemical indices and with lifestyle parameters. Body weight, fat and waist circumference were positively correlated with “Western-type pattern” (*p* = 0.030, *p* = 0.004 and *p* = 0.028, respectively) and “Animal meat and sauces pattern” (*p* = 0.001, *p* = 0.036 and *p* = 0.041, respectively). FFM, TBW and BMI were positively correlated with “Animal meat and sauces pattern” (*p* = 0.004, *p* = 0.012 and *p* = 0.044, respectively). Regarding biochemical parameters, insulin was positively correlated with “Western-type pattern” (*p* = 0.014) and negatively with “Mediterranean-like diet pattern” (*p* = 0.037). HDL was positively correlated with “Mediterranean-like diet pattern” (*p* = 0.011). ALT and AST were positively correlated with “Western-type pattern” (*p* = 0.041 and *p* = 0.029, respectively). Fe was negatively correlated with “Western-type pattern” (*p* = 0.043) and “Animal meat and sauces pattern” (*p* = 0.024). Vitamin D was negatively correlated with “Western-type pattern” (*p* = 0.013) and CRP positively correlated with “Animal meat and sauces pattern” (*p* = 0.010). Finally, lifestyle parameters showed some significant correlations with the four patterns. More specifically, AII was positively correlated with “Western-type pattern” (*p* = 0.042). Rosenberg Self-Esteem scale, PCS-12, MCS-12 were positively correlated (*p* = 0.014, *p* = 0.006 and *p* = 0.038, respectively), whereas CESD-R negatively correlated, with “Mediterranean-like diet pattern” (*p* = 0.033). IPAQ positively correlated with “Mediterranean-like diet pattern” (*p* = 0.023) and negatively with “Animal meat and sauces pattern” (*p* = 0.012).

Then, we applied linear regression models for the associations of dietary patterns with study variables that were significant in the correlation analysis, and, thus, the Healthy pattern was not included in this analysis (Table 4). The first model was unadjusted (β^1^), the second was adjusted for age, gender and BMI (β^2^), the third for age, gender, BMI, physical activity and smoking status (β^3^). Finally, a model adjusted for all the above parameters, plus medication and the scoring for the other three patterns, was applied (β^4^). The “Western-type pattern” showed statistically significant positive association with fat (β^4^ = 4.76, *p* < 0.001), WC (β^4^ = 4.55, *p* = 0.009), insulin (β^4^ = 0.23, *p* = 0.012), ALT (β^4^ = 0.26, *p* = 0.012) and ALP (β^4^= 10.91, *p* = 0.037) and negative association with vitamin D levels (β^4^ = −0.49, *p* = 0.037). The “Mediterranean-like diet pattern” was positively associated with HDL (β = 4.02, *p* = 0.021), and MCS-12 (β^4^ = 0.87, *p* = 0.014) and negatively with CESD-R (β^4^ =−5.52, *p* = 0.008). Finally, the “Animal meat and sauces” pattern was positively associated with TBW (β^4^ = 0.18, *p* = 0.008), FAT (β^4^ = 6.17, *p* = 0.033), FFM (β^4^ = 0.03, *p* = 0.034).

## 4. Discussion

In this study, we investigated the associations between the four main dietary patterns identified in a Greek population of metabolically unhealthy people with obesity and several parameters related to metabolic disorders. To the best of our knowledge, this is the first study in Greece with a holistic approach in analyzing the relationships between anthropometric, biochemical, lifestyle, physical activity, and psychological health parameters with dietary patterns in a metabolically unhealthy study population.

The dietary patterns identified in our cohort were the “Western-type pattern”, the “Mediterranean-like diet pattern”, the “Healthy pattern” and the “Animal meat and sauces pattern”. Similar patterns were previously reported on in cross-sectional studies that investigated dietary patterns associated with the presence of metabolic disorders. For example, the Western-type dietary pattern was found to have a direct association with metabolic syndrome (MS) prevalence in many studies [16,17,18] and higher adherence to a Western dietary pattern was associated with greater risk of developing MS in prospective cohort studies [19,20]. In a meta-analysis conducted by Rodríguez-Monforte et al., [18] which included 28 cross-sectional studies, the pooled odds ratio (OR) for MS was 0.83 for prudent/healthy patterns and 1.28 for Western/unhealthy patterns. In Greece, in the ATTICA study, the adoption of a traditional Mediterranean-like diet [21] and of a healthy food pattern (rich in low-fat products) [22] was associated with lower odds of MS. Finally, “energy-dense” dietary patterns, characterized by higher consumption of animal meat and/or sauces were usually significantly associated with MS [22,23,24].

Regarding the associations with cardiometabolic and other factors related to metabolic disorders, our analysis revealed some interesting findings. More specifically, the “Western-type pattern” was positively associated with fat, WC, insulin, ALT and ALP, and negatively associated with vitamin D. Western or western-like patterns have been previously associated with body weight, body fat, waist circumference and BMI in healthy, obese and patients with metabolic disorders [17,25,26,27,28,29]. In CARDIA, a prospective study with 5115 young adults, individuals following the prudent diet had lower risk of high WC and MS than Western diet consumers [30]. BMI and % body fat were positively associated with an “energy-dense meat” pattern, similar to western patterns in the EXPLORE study, which investigated dietary patterns and body composition profiles in premenopausal New Zealand European women [31].

Western or “westernized” dietary patterns have also been associated with several biomarkers of obesity and CVD risk. One of the first studies in this field was The Health Professionals Follow-up study, a prospective cohort study of 51529 US male health professionals, which revealed positive correlations of the Western pattern with insulin and negative with plasma folate concentrations [32]. Additionally, the Western pattern was positively associated with total cholesterol, insulin and fasting blood glucose in patients with T2DM [33]. In the NHANES analysis, which included dietary patterns determined in 13310 US adults, the Western pattern was associated positively with serum insulin, and glycated hemoglobin and negatively with red blood cell folate concentrations [34]. In our study, a positive association with insulin levels confirmed the importance of diet quality in insulin resistance; as such, it is well established that lower consumption of fruits and vegetables and higher consumption of refined grains, processed meat and sweets are associated with increased risk of T2DM [35]. We also observed a positive association with liver function enzymes (AST and ALT). Individuals with high adherence to a Western dietary pattern, were more likely to have elevated ALT and AST levels, not only in MS, but in the general population as well [36,37]. Finally, the Western-type pattern was inversely associated with vitamin D levels. Previous studies have shown that dietary patterns rich in dairy, sea food, eggs, and vegetables were positively associated with serum 25(OH)D levels, while patterns rich in sweets, alcohol, fats, and soft drinks were inversely associated with serum 25(OH)D [38,39,40].

In our study, the “Mediterranean-like diet pattern” was positively associated with HDL and MCS-12 and negatively with CESD-R. In the ATTICA study. A pattern rich in fish, vegetables, legumes, cereals, and fruits, comparable to our “Mediterranean-like pattern”, was inversely associated with HDL-cholesterol level [22]. Nevertheless, there is a plethora of studies showing that greater adherence to the Mediterranean diet is associated with improved blood lipid profile and a significant reduction of major cardiovascular events of almost 30% [41,42].

MCS-12 and CESD-R are two very useful tools for validating mental health and depression, respectively, and, when combined, they indicate an excellent convergent validity in identifying probably clinically significant depression [43]. Excess body weight expressed as disarrayed metabolic status and depression are linked through inflammation and stress in a bidirectional way [44,45]. This vicious cycle is sustained not only through the obvious severe impact of both pathologies on one’s mental health but also through intertwined biochemical pathways [44]. Herein, the “Mediterranean-like diet pattern” was positively associated with MCS-12 and negatively with CESD-R, indicating better mental health for those with a greater adherence to this dietary pattern. Similarly, in the MARK study, a longitudinal study of 500 Spanish people with intermediate cardiovascular risk, greater adherence to the Mediterranean diet was associated with higher scores on the MCS-12 [46]. In a large cohort of North Americans with osteoarthritis, a higher adherence to the Mediterranean diet was associated with a higher PCS-12 and a lower CESD [47]. The same did not occur in the Seniors-ENRICA cohort. The PREDIMED score and Trichopoulou’s Mediterranean Diet score (MSD) were used to measure Mediterranean diet adherence. Only PREDIMED was associated with PCS, whereas neither PREDIMED nor MSD was associated with PCS or MCS [48]. An improved nutritional status may not be the only factor contributing to mental health when adherence to the Mediterranean diet is high. Improved mental health may also be due to a Mediterranean lifestyle that not only encourages healthy food choices but also enables friends and family to share lunchtimes, which contributes to a higher quality of life [49].

Finally, the “Animal meat and sauces pattern” was positively associated with TBW (kg), BF (kg) and FFM (kg). FFM consists of metabolically active tissues, bones, organs and TBW. The association of this pattern with both parameters was apparently due to the fact that high protein content of animal origin in the diet increases muscle mass and high muscle mass parallels high TBW due to the high content of intracellular water. On the other hand, the association of this pattern with BF could be explained by the high fat content in the diet.

This study contained several strengths, one being the use of a validated food frequency questionnaire, providing comprehensive information on eating habits, together with interviewing of the participants by experienced nutritionists and adjustment for strong potential confounders in the regression analyses. Furthermore, it was conducted in a well-characterized Greek population exploring the relationship between different dietary patterns of this Mediterranean country and health outcomes related to metabolic disorders with a statistical approach that enabled the evaluation of the overall quality of the diet [50]. Limitations might include possible under- or over- reporting of FFQs, the relatively small sample size, the fact that associations derived from an observational study did not necessarily indicate causality, and confirmation of the results in prospective studies was needed, and the disadvantages that sprout from the nature of the PCA and EFA analyses [50].

## 5. Conclusions

In conclusion, our results suggest that dietary patterns characterized by high consumption of energy-dense and animal derived foods are positively associated with anthropometric and biochemical parameters related to metabolic disorders. On the contrary, plant-based, healthier dietary patterns are associated with a better biochemical and mental health profile of metabolically unhealthy obese individuals.

## Figures and Tables

**Table 1:** Descriptive characteristics of the study population.

Characteristics of the Study Participants	
Sex, *n* (%)	
Men	55 (37.7)
Women	91 (62.3)
Age (years), mean (SD)	53.5 (11.4)
Educational years, mean (SD)	15.3 (3.4)
Family status, *n* (%)	
Married	109 (74.6)
Divorced	7 (4.8)
Single	20 (13.7)
Widowed	5 (3.4)
Other	5 (3.4)
Smoking, *n* (%)	
No	109 (74.7)
Yes	33 (22.6)
N/A	4 (2.7)
Antihypertensive treatment, *n* (%)	
No	91 (62.3)
Yes	55 (37.7)
Statins, *n* (%)	
No	104 (71.2)
Yes	42 (28.8)
Antidiabetic agents, *n* (%)	
No	111 (76.0)
Yes	35 (24.0)
Anthropometric parameters	
ΒΜΙ (kg/m^2^), mean (SD)	34 (6.3)
WC (cm), mean (SD)	110.4 (13.4)
HC (cm), mean (SD)	117.9 (14.9)
Lifestyle parameters	
IPAQ-SF (total MET- min/week), mean (SD)	1723 (2142)
AII, mean (SD)	5.4 (3.7)
CESD-R, mean (SD)	16 (10)
Rosenberg Self-Esteem scale, mean (SD)	31 (5)
PCS-12, mean (SD)	45.7 (9.1)
MCS-12, mean (SD)	48.6 (9.5)

Data are expressed as counts (%) or mean values (standard deviation, SD). BMI, body mass index; WC, waist circumference; HC, hip circumference; IPAQ-SF, International Physical Activity Questionnaire Short Form; AII, Athens Insomnia Scale; CESD-R, Center for Epidemiologic Studies Depression Scale Revised; PCS-12, Physical Component Score; MCS-12, Mental Composite Score.

**Table 2:** Food groups and respective factor loadings for the four dietary patterns of the study.

	Dietary Patterns			
Food Groups	Western-Type Pattern	Mediterranean-Like Diet Pattern	Healthy Pattern	Animal Meat and Sauces Pattern
Dairy (High-fat)	0.57			
Dairy (Low-fat)			0.59	
Refined grains	0.66			
Whole grains		0.60		
Fast Food	0.74			
Red Meat				0.50
Processed Meat	0.57			
Fish		0.59		
Vegetables|cooked mixed vegetables		0.75		
Fruits|fruit juices		0.53		
Pies	0.66			
Sweets	0.56			
Salty Snacks	0.63			
Olive oil | olives			0.63	
Soft drinks	0.52			
Sauces				0.43
Animal & HydrogenatedFats	0.54			
Poultry				0.70
Pulses		0.50		
Dried fruits		0.62		
Nuts		0.66		
Coffee and Tea			0.55	
Seed oil	0.53			
Alcohol			−0.29	
Eggs				0.29
*Cronbach’s a*	0.79	0.82	0.71	0.73

**Table 3:** Correlation between dietary patterns and anthropometrics, biochemical and lifestyle parameters.

Parameters		Western-Type Pattern	Mediterranean-Like Diet Pattern	Healthy Pattern	Animal Meat and Sauces Pattern
Anthropometrics					
BW (kg)	rho	0.19	−0.01	0.02	0.29
	*p*	**0.030**	0.947	0.801	**0.001**
BF (%)	rho	0.13	0.06	0.06	0.02
	*p*	0.136	0.470	0.504	0.844
BF (kg)	rho	0.26	0.07	0.06	0.19
	*p*	**0.004**	0.457	0.499	**0.036**
FFM (kg)	rho	0.05	−0.11	−0.05	0.26
	*p*	0.565	0.246	0.616	**0.004**
TBW (kg)	rho	0.11	−0.05	−0.08	0.23
	*p*	0.226	0.579	0.355	**0.012**
ΒΜΙ (kg/m^2^)	rho	0.13	0.04	0.08	0.18
	*p*	0.130	0.687	0.390	**0.044**
WC (cm)	rho	0.20	−0.08	0.03	0.18
	*p*	**0.028**	0.381	0.747	**0.041**
HC(cm)	rho	0.14	0.09	0.06	0.10
	*p*	0.102	0.312	0.470	0.236
Biochemical					
Urea (mg/dL)	Rho	−0.04	−0.11	−0.09	0.10
	*p*	0.648	0.214	0.284	0.265
Uricacid (mg/dL)	Rho	−0.09	0.09	0.03	0.04
	*p*	0.295	0.289	0.726	0.693
Creatinine (mg/dL)	Rho	−0.02	−0.12	−0.02	0.13
	*p*	0.863	0.190	0.805	0.159
Glucose (mg/dL)	Rho	−0.03	−0.13	0.09	−0.08
	*p*	0.752	0.151	0.337	0.349
Insulin (μIU/mL)	Rho	0.23	−0.20	0.00	0.11
	*p*	**0.014**	**0.037**	0.964	0.236
TC (mg/dL)	Rho	0.11	−0.14	−0.08	−0.03
	*p*	0.220	0.119	0.373	0.768
TG (mg/dL)	Rho	0.12	−0.12	−0.02	−0.05
	*p*	0.190	0.168	0.817	0.603
HDL (mg/dL)	Rho	−0.10	0.22	0.02	−0.09
	*p*	0.281	**0.011**	0.789	0.331
LDL (mg/dL)	Rho	0.10	0.11	−0.10	−0.04
	*p*	0.274	0.205	0.270	0.665
AST (iu/L)	Rho	0.00	0.04	0.05	−0.02
	*p*	0.997	0.625	0.588	0.819
ALT (iu/L)	Rho	0.19	0.02	0.13	0.06
	*p*	**0.041**	0.843	0.131	0.492
γ-GT (iu/L)	Rho	0.16	−0.09	0.03	0.11
	*p*	0.090	0.338	0.764	0.227
ALP (U/L)	Rho	0.20	0.05	−0.01	−0.02
	*p*	**0.029**	0.576	0.925	0.825
Fe (μg/dL)	Rho	−0.19	0.02	−0.05	−0.21
	*p*	**0.043**	0.831	0.579	**0.024**
Ferritin (ng/mL)	Rho	0.06	0.09	0.00	0.05
	*p*	0.538	0.316	0.991	0.556
Albumin (g/dL)	Rho	0.08	0.14	0.05	−0.08
	*p*	0.390	0.131	0.568	0.404
Vitamin D (ng/mL)	Rho	−0.22	0.01	−0.07	−0.10
	*p*	**0.013**	0.917	0.433	0.254
CRP (mg/L)	Rho	0.08	0.09	0.04	0.24
	*p*	0.409	0.326	0.655	**0.010**
LDH (U/L)	Rho	0.04	0.07	0.06	0.03
	*p*	0.712	0.433	0.555	0.719
Lifestyle					
AII	Rho	0.19	−0.14	−0.12	0.17
	*p*	**0.042**	0.120	0.205	0.059
CESD-R	Rho	0.17	−0.21	0.00	0.09
	*p*	0.088	**0.033**	0.991	0.340
Rosenberg Self-Esteem scale	Rho	−0.07	0.24	0.07	0.12
	*p*	0.482	**0.014**	0.443	0.212
PCS-12	Rho	−0.03	0.26	−0.07	0.03
	*p*	0.785	**0.006**	0.444	0.759
MCS-12	Rho	−0.15	0.20	0.04	−0.12
	*p*	0.121	**0.038**	0.679	0.200
IPAQ-SF (total MET- min/week)	Rho	0.03	0.20	0.05	−0.22
	*p*	0.748	**0.023**	0.567	**0.012**

Values resulted from Spearman test. Level of significance was set as 0.05. Significant *p* are in bold. BW, body weight; BF, body fat; FFM, free fat mass; TBW, total body water; BMI, body mass index; WC, waist circumference; HC; hip circumference; TC, total cholesterol; TG, triglycerides; HDL, high-density lipoprotein; LDL, low-density lipoprotein; AST, aspartate aminotransferase; ALT, alanine aminotransferase; γ-GT, γ-glutamyl transferase; ALP, alkaline phosphatase; Fe, iron; CRP, C-reactive protein; LDH, lactate dehydrogenase; AII, Athens Insomnia Scale; CESD-R, Center for Epidemiologic Studies Depression Scale Revised; PCS-12, Physical Composite Score; MCS-12, Mental Composite Score; IPAQ-SF, International Physical Activity Questionnaire Short Form.

**Table 4:** Association between dietary patterns scores and study parameters.

	Western-Type Pattern							
Dependent Variables	β^1^ (SE)	*p*	β^2^ (SE)	*p*	β^3^ (SE)	*p*	β^4^ (SE)	*p*
BF (kg)	9.01 (2.55)	**0.001**	5.87 (1.11)	**<0.001**	5.66 (1.14)	**<0.001**	4.38 (1.26)	**0.001**
WC(cm)	6.19 (2.99)	**0.041**	5.13 (1.56)	**0.001**	5.42 (1.60)	**0.001**	4.95 (1.77)	**0.006**
Insulin (μIU/mL)	0.21 (0.09)	**0.016**	0.23 (0.09)	**0.009**	0.23 (0.09)	**0.009**	0.24 (0.09)	**0.008**
ALT (iu/L)	0.20 (0.10)	0.035	0.24 (0.10)	**0.014**	0.24 (0.10)	**0.017**	0.25 (0.10)	**0.019**
ALP (U/L)	9.91 (4.35)	**0.025**	8.83 (4.68)	**0.062**	9.55 (4.75)	**0.047**	9.78 (4.97)	**0.0** **41**
Fe (μg/dL)	−0.36 (0.18)	**0.040**	−0.27 (0.2)	0.176	−0.28 (0.2)	0.174	−0.19 (0.22)	0.393
Vitamin D (ng/mL)	−0.41 (0.2)	**0.046**	−0.44 (0.21)	**0.043**	−0.44 (0.21)	**0.041**	−0.55 (0.23)	**0.021**
AII	1.99 (0.85)	**0.021**	1.51 (0.87)	0.085	1.42 (0.91)	0.121	1.10 (0.98)	0.263
	Mediterranean-like diet pattern							
Insulin (μIU/mL)	−0.17 (0.09)	**0.050**	−0.17 (0.09)	**0.050**	−0.16 (0.09)	0.070	−0.16 (0.10)	0.102
HDL (mg/dL)	4.34 (1.7)	**0.012**	3.86 (1.5)	**0.011**	3.44 (1.53)	**0.026**	4.03 (1.73)	**0.022**
CESD-R	−4.13 (1.9)	**0.032**	−3.86 (1.88)	**0.043**	−4.09 (1.92)	**0.036**	−5.63 (2.07)	**0.008**
Rosenberg Self-Esteem scale	0.19 (0.08)	**0.023**	0.18 (0.08)	**0.034**	0.19 (0.08)	**0.020**	0.28 (0.35)	0.417
PCS-12	0.22 (0.08)	**0.009**	0.18 (0.08)	**0.025**	0.18 (0.08)	**0.031**	0.53 (0.34)	0.124
MCS-12	0.19 (0.08)	**0.026**	0.19 (0.08)	**0.023**	0.19 (0.08)	**0.025**	0.87 (0.35)	**0.016**
	Animal meat and sauces pattern							
BF (kg)	8.09 (2.48)	**0.001**	3.54 (1.21)	**0.004**	4.01 (1.22)	**0.001**	6.17 (2.88)	**0.033**
FFM (kg)	0.04 (0.02)	**0.048**	0.02 (0.01)	**0.033**	0.02 (0.01)	**0.040**	0.03 (0.02)	**0.034**
TBW (kg)	0.20 (0.09)	**0.033**	0.15 (0.06)	**0.016**	0.16 (0.06)	**0.010**	0.17 (0.07)	**0.011**
ΒΜΙ (kg/m^2^)	2.63 (1.27)	**0.041**	2.24 (1.40)	0.113	2.47 (1.44)	0.089	2.23 (1.52)	0.144^5^
WC (cm)	5.74 (2.89)	**0.049**	3.78 (1.60)	**0.020**	4.33 (1.63)	**0.009**	2.89 (1.74)	0.100
Fe (μg/dL)	−0.42 (0.2)	**0.041**	−0.41 (0.22)	0.067	−0.42 (0.23)	0.067	−0.40 (0.25)	0.114
CRP (mg/L)	2.26 (1.13)	**0.048**	2.21 (1.26)	0.082	2.23 (1.3)	0.089	2.09 (1.43)	0.146

^1^ unadjusted regression coefficient of each pattern (Standard Error), ^2^ regression coefficients of each pattern adjusted for age, gender and BMI (Standard Error), ^3^ regression coefficients of each pattern adjusted for age, gender, BMI, physical activity and smoking (Standard Error), ^4^ regression coefficients of each pattern adjusted for age, gender, BMI, physical activity, smoking, current medication and the other three patterns (Standard Error). ^5^ In this analysis, BMI was not included as independent variable. Values resulted from linear regression models. Level of significance was set as 0.05. Significant *p* are in bold. BF, body fat; FFM, free fat mass; TBW, total body water; WC, waist circumference; ALT, alanine aminotransferase; ALP, alkaline phosphatase; Fe, iron; HDL, high density cholesterol; CRP, C-reactive protein; AII, Athens Insomnia Scale; CESD-R, Center for Epidemiologic Studies Depression Scale Revised; PCS-12, Physical Composite Score; MCS-12, Mental Composite Score.

## Data Availability

The data presented in this study are available on request from the corresponding author.

## Supplementary Materials

The following supporting information can be downloaded at: https://www.mdpi.com/article/10.3390/nu14235064/s1, Table S1: Food groups and food items
