# Supplementary material for: Dietary Patterns, Cardiometabolic and Lifestyle Variables in Greeks with Obesity and Metabolic Disorders
Source: Nutrients. 2022 Nov 28;14(23):5064. doi: 10.3390/nu14235064 (PMC9738070; doi:10.3390/nu14235064)
Supplement: Supplementary file 1 [file nutrients-14-05064-s001.zip › nutrients-2040257-supplementary.pdf]

nutrients

MDPI

Supplementary Table S1. Food groups and food items.

	Food Groups	Food Items (69)
**1**	Dairy (High-fat)	High fat milk/yogurt ǀ Yellow cheese/cream cheese ǀ White cheese (e.g. feta cheese) ǀ Ice-cream/milk-shake/cream/rice pudding
**2**	Dairy (Low-fat)	Low fat milk/yogurt ǀ Low fat cheese (light/ cottage)
**3**	Eggs	
**4**	Refined grains	White bread/toast ǀ white rice ǀ Pasta/ pearl barley ǀ Potatoes boiled/baked/mashed
**5**	Whole grains	Whole meal bread/rusk ǀ brown rice ǀ Whole meal pasta
**6**	Fast Food	Toasted sandwich/sandwich ǀ burger-bread ǀ French fried potatoes
**7**	Red Meat	Veal (steak, filet) ǀ Burger/ meat balls/ minced-meat ǀ Pork (steak, filet) ǀ Lamb/goat/game/ lamb-chops ǀ Pastitsio/moysakas/papoytsakia
**8**	Processed Meat	Sausage / bacon ǀ Light/no fat cold sliced meats ǀ Cold sliced meats
**9**	Poultry	Chicken/turkey (all kind)
**10**	Fish	Small fish ǀ large fish ǀ sea-food (octopus, sleeve-fish, prawns)
**11**	Pulses	Pulses (lentils, beans, chickpeas)
**12**	Vegetables ǀ cooked mixed vegetables	Tomato/cucumber/carrot/pepper ǀ Broccoli/cauliflower/courgetti ǀ Lettuce/cabbage/spinach/rocket ǀ Greens/celery/spinach ǀ Spinach-rice/ cabbage-rice ǀ Petit pois (peas) / green beans / okra /artichoke
**13**	Fruits ǀ fruit juices	Orange ǀ Apple/pear ǀ Other winter-fruits ǀ banana ǀ Other summer – fruits ǀ fruit juice
**14**	Dried fruits	
**15**	Nuts	
**16**	Pies	Home made pies (e.g. Cheese-pie, spinach-pie) ǀ Pies
**17**	Sweets	Sweets made in tray ǀ Sweet preserves/stewed fruit/fruit – jelly ǀ Gateau/tart ǀ Croissant/gofer/cake/biscuits ǀ chocolate ǀ Honey/marmalade/sugar ǀ Cereal/cereal bars
**18**	Salty Snacks	Chips/pop-corn ǀ crisp bread
**19**	Olive oil ǀ olives	
**20**	Alcohol	Wine ǀ Beer ǀ other alcohol
**21**	Soft drinks	Soft drinks ǀ Light soft drinks
**22**	Coffee and Tea	Coffee, Tea/other teas
**23**	Sauces	Mayonnaise/sauce ǀ Light mayonnaise/light sauce
**24**	Seed oil	
**25**	Animal & Hydrogenated Fats	Butter ǀ margarine
